# Chemical Composition and Biological Activity of *Salvia officinalis* L. Essential Oil

**DOI:** 10.3390/plants12091794

**Published:** 2023-04-27

**Authors:** Zvonimir Jažo, Mateo Glumac, Vlatka Paštar, Sanida Bektić, Mila Radan, Ivana Carev

**Affiliations:** 1 Department of Biochemistry, Faculty of Chemistry and Technology, University of Split, Ruđera Boškovića 35, 21000 Split, Croatia; zvonimir.jazo@gmail.com (Z.J.); mateo.glumac@mefst.hr (M.G.);; 2 Regional Laboratory Split, Croatian Veterinary Institute, Poljička Cesta 33, 21000 Split, Croatia; 3 School of Medicine, University of Split, Šoltanska 2, 21000 Split, Croatia; 4 Mediterranean Institute for Life Science, Meštrovićevo Šetalište 45, 21000 Split, Croatia; 5 Faculty of Sciences, University of Tuzla, Univerzitetska 4, 75000 Tuzla, Bosnia and Herzegovina; sanida.osmanovic@untz.ba; 6 NAOS Institute of Life Science, 355, Rue Pierre-Simon Laplace, 13290 Aix, France

**Keywords:** breast cancer cell lines, cell viability, human dermal fibroblast, lung cell lines, MTS assay, *Salvia officinalis* L., SEM

## Abstract

In our study, we investigated the chemical composition and cytotoxic activity of essential oils isolated from Dalmatian sage (*Salvia officinalis* L.) collected along the Adriatic coast of Croatia. Scanning electron microscopy (SEM) was used to examine the morphology of the stem and leaf surfaces. Essential oil excretory glands were detected on both the leaves and stem surfaces. The essential oils were isolated by hydrodistillation, and their chemical composition was determined by gas chromatography and mass spectrometry (GC-MS). Sage essential oils were mixtures of terpene compounds, among which the most common were: *α*- and *β*-thujone, camphor, and 1,8-cineol. Cytotoxic activity was tested using MTS assay on multiple cell lines: normal and immortalized fibroblasts (HF77FA and HDF-Tert), immortalized lung line (BEAS-2B), and breast adenocarcinoma (MDA-MB-231). The growth of treated cells was determined relative to control conditions without treatment. The immortalized lung line was the least resistant to the activity of the essential oils, whereas immortalized fibroblasts were the most resistant. Statistical analysis has connected the cytotoxic effect and chemical composition of the studied essential oils. To the best of our knowledge, this work is the first testing of the cytotoxic activity of *S. officinalis* EO’s on the BEAS-2B, HF77FA, and HDF-Tert cell lines. The presented data on essential oil chemical composition and cytotoxic effect on 4 types of human cells supports pharmacotherapeutic potential this plant is known to have.

## 1. Introduction

The genus Salvia includes around 900 plant species and is one of the main genera of the *Lamiaceae* family [1]. *Salvia officinalis* L. is an evergreen, bushy, up to 60 cm high plant with woody stems and hairy leaves with violet-blue, pink, or white flowers [2]. The indigenous habitats of *S. officinalis* L. are located on the coastal area and islands along the Adriatic and Ionian seas, Mediterranean, and inland areas of southern Serbia. Western Macedonia and Greece [3].

Because of its medicinal properties, *S. officinalis* has been used for therapeutic purposes in folk medicine for centuries [1,2,4]. The pharmacological properties of *S. officinalis* extracts originate from its complex chemical composition, which contains a variety of bioactive compounds, such as flavonoids, phenolic acids, terpenes, and others [5]. The biological properties of *S. officinalis* essential oils (EO) are mainly associated with *α*- and *β*-thujones, camphor, and 1,8-cineol [6]. The quantity of the mentioned compounds determines their market value [7]. Sage Eos with an amount of thujone greater than 30% and an amount of camphor less than 20% have a higher value on the market [2].

According to the chemical definition, EOs are complex mixtures of volatile organic compounds (VOC) produced as secondary plant metabolites to protect against herbivores, insects, and microorganisms. They enable communication between plants, as well as responses to various environmental stimuli and could be isolated from the whole plant or one of its parts (seed, root, stem, leaf, flower) depending on the plant species [8,9].

Essential oils are produced and stored in specialized structures such as secretory glands, cavities, channels, and glandular trichomes, which are morphological glandular structures of epidermal origin that can be found on the stem and leaf surfaces [10]. They are specific for the plant parts with hydrophobic secretions that produce EOs [1]. Glandular trichomes are characteristic of the *Lamiaceae* family and contain a very short stem and a head composed of many rosette-like cells covered by a joint cuticle. The head cells secrete an EO that accumulates under the cuticle, which causes the cuticle to rise and form a vast subcuticular space in the shape of a calotte [11,12,13,14]. Non-glandular trichomes are filamentous, unbranched, long, and usually more concentrated around conductive bundles, forming a woolly coating on the leaf’s surface that represents a protective barrier to the external environment [15]. Epidermal trichomes found on the surface of most plants can serve as the first line of defense against herbivores and pathogens, resistance to pests, protection from UV-B radiation, effects of the environment, drying, and evaporation [16,17,18,19,20,21,22,23,24]. It has been shown that the number of peltate glandular trichomes and volume of glandular trichomes positively correlate with the EO yield [10,25]. Plant glands on the tissue surface can be detected by scanning electron microscopy (SEM) in high resolution following the protocol that preserves the natural structure of the tissue surface [26].

The chemical composition of *S. officinalis* EO on the market is regulated by the ISO 9909 standard, which prescribes the recommended amounts for eleven compounds: *α*-pinene (1.0–6.5%), camphene (1.5–7%), limonene (0.5–3%), 1,8-cineole (5.5–13%), sum of linalool and linalyl-acetate (≤1%), *α*-thujone (18–43%), *β*-thujone (3.0–8.5%), camphor (4.5–24.5%), bornyl acetate (≤2.50%), and *α*-humulene (≤12%). Physiological and pharmacological purposes require an adequate amount of an individual compound, as a misbalance in the composition could cause a loss of medicinal properties or toxic effects [27].

The cytotoxic activity of *S. officinalis* EO was studied previously on multiple cell lines: cervix (HeLa), mammary gland (MCF7), prostate (LNCaP) [28] human melanoma cell lines (A375, M14, and A2058) [29], human lung cancer cell lines (A549, NCI-H226) [30], human umbilical vein endothelial cells (HUVEC), human chronic myeloid leukemia (K562), and mouse fibrosarcoma (Wehi) [31], yet authors usually fail to consider the relationship between EO composition and its cytotoxic activity. Due to their biological activity, EOs, a mixture of natural compounds, are important in research for finding new drugs with biological effects. Therefore, studies linking EOs medicinal properties with its chemistry are particularly interesting, especially investigations between organic compounds’ structure and biological activity [32].

This study aimed to investigate the relationship between the chemical composition of seven representatives of *S. officinalis* EOs and their cytotoxic properties on healthy and cancerous cell lines. We applied several chemical classification strategies to better characterize bioactive compounds in the complex EO mixtures. In addition, we examined the structures of the stem and leaf tissues of *S. officinalis* using SEM.

## 2. Results and Discussion

### 2.1. Salvia officinalis Leaf and Stem Morphology

**Figure 1:**
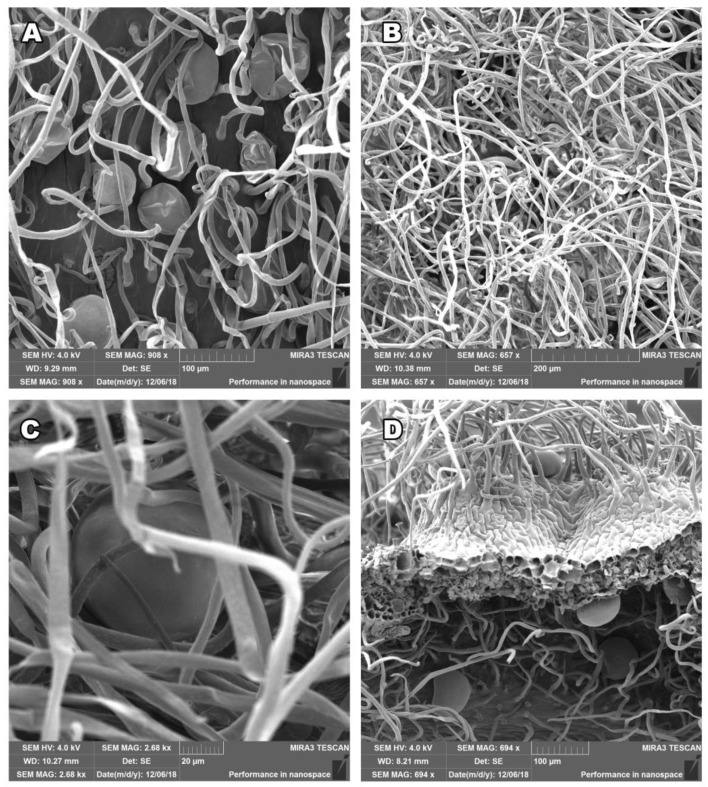
*Salvia officinalis* L. stem surface (**A**,**C**) and leaf (**B**,**D**) images, created using a scanning electron microscope (SEM). The densely arranged epidermal hairs (trichomes) and oil glands can be observed in (**A**,**B**). Non-glandular trichomes are more numerous, simple, and mostly thread-like, they are upright or slightly inclined towards the epidermis in (**C**,**D**). The fixative was glutaraldehyde for (**A**,**B**) and formaldehyde for (**C**,**D**). Scale bars of 20 µm, 100 µm, and 200 µm are shown.

**Figure 2:**
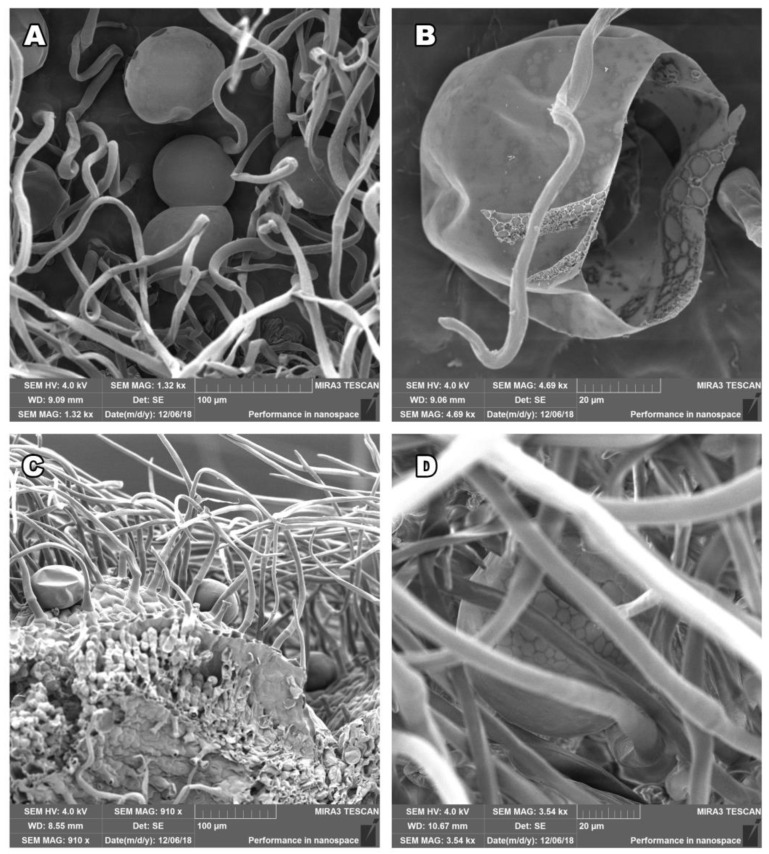
*Salvia officinalis* interconnected plant glands (**A**) where the synthesis and accumulation of EOs occur. (**B**) an open plant gland after the release of EO into the environment. (**C**) A side view where a cross-section was made at the site of the disrupted plant structure. (**D**) The leaf surface is covered with glandular and dense non-glandular trichomes, allowing the plant to withstand environmental challenges during its active life cycle. The fixative used was glutaraldehyde for (**A**,**B**,**D**) and formaldehyde for (**C**). Scale bars of 20 µm, 100 µm, and 200 µm are shown.

To study stem and leaves tissue structures in *S. officinalis* we have used scanning electron microscopy (SEM) in high resolution following two protocols for sample preparation and fixation: glutaraldehyde and FAA fixation, with the intention to preserve the natural structure of the tissue surface. The photos taken show the actual state of the plant immediately before the distillation process, the surface examination was carried out with dry plant material. Our fixation approaches successfully preserved plant structures as shown in Figure 1 and Figure 2. The surface of aerial parts of *S. officinalis,* as observed using SEM in our study, contained glandular structures of different morphologies of epidermal origin. Trichomes originate from epidermal cells, arising exclusively from epidermal meristemoids, and as per rule: from one initial cell [11,12]. They are generally considered to be the site of biosynthesis or accumulation of EOs [15]. The observed structures excrete hydrophobic secretions, resins, and terpene compounds as part of the EOs [1]. The secretion system of *S. officinalis* consists of two types of glandular trichomes: peltate and capitate, which can be distinguished by the size of the head and the length of the stalk. There is one exception when the capitate glandular trichome in *Salvia officinalis* L., was described as having a head resembling a volcano crater [33].

The non-glandular trichomes, with air replacing the protoplasm, are numerous, articulated, and bent, covering trichomes with narrow, elongated cells. The glandular trichomes could be of the lamiaceous type, with a unicellular stalk and an 8- to 12-celled head-covered joint cuticle; tiny glandular trichomes with a unicellular or multicellular stalk and a unicellular head, usually on an epidermis; or more rarely, glandular trichomes with a unicellular stalk and a bicellular head. Our leaf and stem morphology study observed, using SEM, densely distributed epidermal hairs and trichomes in both the stem and leaf epidermis (Figure 1A,B). Further, the stem and leaf surface were covered by non-glandular (dead) and glandular (live) trichomes located on the upper and lower epidermal surfaces (Figure 1C,D). Figure 1D and Figure 2C show the three-dimensional structure, and the visible deformation that was created at the cutting point.

The glands containing EO can be interconnected, as observed in Figure 2A. This can lead to the conclusion that the accumulated EO is released by spraying the cuticle (Figure 2B). The leaf’s surface has glandular and non-glandular trichomes (Figure 2C,D). We could also observe that the leaf surfaces were rich in non-glandular and glandular trichomes, which enables the plants to withstand environmental challenges during their active life cycle [34].

Our study was focused on describing the presence of glandular trichomes on the steam surface. For the Lamiace species, it has been shown that the presence of plant glands on the surface of plant organs correlates with the EO yield [10,25]. Our SEM figures prove that the glandular trichomes are present on stem surfaces, which can affect EO production as both stems and leaves could be used to isolate the oil, improving the yield and reducing the biological waste created in the process. However, a further study involving a more significant number of samples is necessary.

### 2.2. Chemical Composition of S. officinalis Essential Oils

Further steps in our study were focused on *S. officinalis* essential oil’s biological activity, for which EO chemical composition was crucial for further analysis and comparison. Therefore, we have analyzed, using GC/MS analysis, seven EOs, isolated by hydrodistillation, from plant material collected from different locations in the Dalmatia region of Croatia (Table 1). We have used homogenous collection conditions of EOs, regarding plant development, environmental factor, and collecting conditions to allow a more consistent comparison of biological activity.

The yield of studied EO’s isolated with hydrodistillation from dried plant material (leaves and stems) was in the range of 0.56–2.14%. Seven EOs had in total 25 identified compounds, which represented 89.2–99.1% of each oil. The chemical compounds found in a high amount in EO’s were common *S. officinalis* chemical constituents: *α*-thujone (12.6–42.6%), *β*-thujone (1.3–13.8%), camphor (12.4–33.3%), and 1,8-cineole (4.2–9.9%). Other compounds that appear in all samples, greater than 2%, are viridiflorol, borneol, and camphene. In only three samples, diterpene compound manool (SO-SV, SO-MA, and SO-LO) was identified in range 1.2–3.8%. According to the literature data, manool in other sage EOs was up to 20% [3].

Chemical compounds found in EOs were classified according to terpene composition, functional groups, compound structure, number of oxygen atoms, and number of unsaturated bonds, the results are presented in Table 2. The obtained classification was used for statistical analysis.

When analyzed statistically, the chemical composition of seven analyzed EOs did not show significant variation, according to ANOVA analysis (*p* > 0.05). Significant variations (ANOVA *p* < 0.0001) were observed when comparing terpene composition, functional groups, amount of cyclic compounds, presence of heteroatom, and the number of unsaturated bonds in analyzed EOs. According to statistical analysis, *S. officnalis* preferably produces EOs composed of unsaturated bicyclic terpenes with a ketone functional group. Differences in yield and chemical composition of EOs isolated from aromatic plants of the same species can result from the influence of different external factors. Environmental conditions in which the plant grew, dry periods during the vegetative phase, water deficit, exposure to the sun, microclimate conditions, climate conditions, salt stress, and other conditions can influence the production and chemical composition of EOs [35,36,37]. We did not make a detailed scientific study of the influence of environmental conditions on EO chemical composition, but we have observed some trends. Plants collected near the sea at the locations Seget Vranjica (SO-SV), Hvar (SO-HV), Marina (SO-MA), and Kornati (SO-KO) gave a higher yield (>1.5%) of EO. This could be due to environmental conditions such as high salt stress, high insulation, and microclimate conditions, the plants were exposed due to the vicinity of the sea [38].

Chemotyping of *S. officinalis* EOs can be done in several ways according to literature data. Considering the amounts of main compounds in sage Eos, we have recognized five chemotype groups [39]. Four samples (SO-SP, SO-LA, SO-HV, and SO-SV) can be classified as chemotype V, having compounds in this order: *α*-thujone > camphor > *β*-thujone > 1,8-cineole. The sample SO-KO can be classified as chemotype I, with an order of compounds: camphor > *α*-thujone > 1,8-cineole > *β*-thujone. For two samples (SO-MA and SO-LO), according to the same classification, it was impossible to determine the chemotype because the order of the main compounds, in terms of amount, does not correspond to any of the proposed chemotype groups. According to another classification strategy, chemotype groups were proposed considering the amount of *α*-thujone, *β*-thujone, and camphor after investigating the EO of *S. officinalis* isolated from the 25 populations in Croatia and Bosnia and Herzegovina [3]. According to this classification strategy, six of the analyzed samples were chemotype A (high amount of *α*-thujone); the exception was the SO-KO sample which is chemotype C (high camphor content, *β*-pinene, borneol, and bornyl acetate).

According to ISO 9909 standard [27] only two samples (SO-MA and SO-LO) meet the composition requirements. All studied *S. officinalis* EOs contained the recommended amount of *α*-pinene and *α*-humulene. The largest differences in the chemical composition from the recommended values were observed in the SO-KO sample, isolated from the plant material from the island of Kornat: a slightly increased amount of camphene (7.4%), the amount of limonene was 3%, which is the upper recommended limit, compared to other samples it contained a lower amount of *α*-thujone (12.6%) and *β*-thujone (1.3%), while the amount of camphor (33.3%) and boronyl acetate (3.3%) exceeded upper recommended limit. Compared to the recommended value, an increased proportion of *β*-thujone was observed in EOs: SO-SP (13.8%), SO-LA (9.5%), and SO-SV (12.2%), while a lower value of 1,8-cineole was observed in the SO-HV sample (4.2%), and the SO-SP sample contains bornyl acetate (3.3%) in an increased amount. The observed differences, compared to ISO 9909 standard could be explained with the effect of collection period [27] and environmental factors present in different locations [29,37].

### 2.3. The Cytotoxicity of S. officinalis Essential Oils

**Figure 3:**
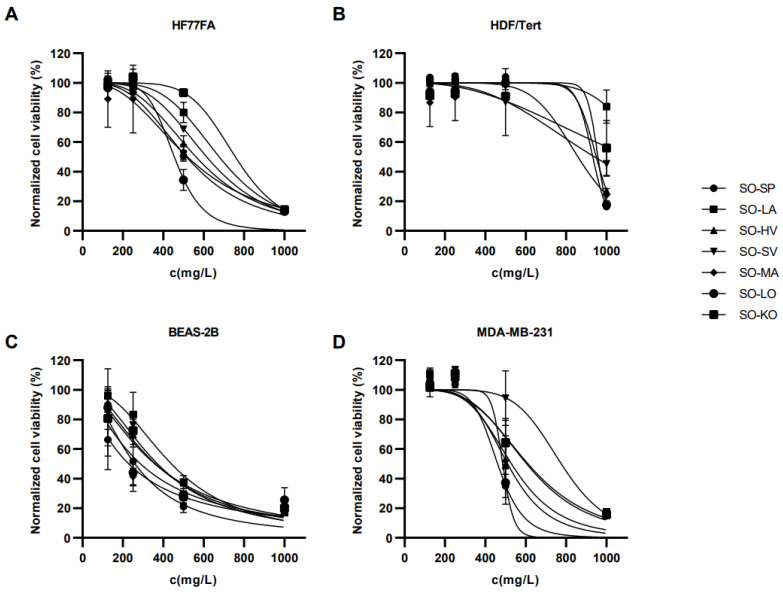
Normalized curves for cell viability, determined comparing the treated and non-treated cell (**A**) normal dermal fibroblasts (HF77FA), (**B**) immortalized human dermal fibroblasts (HDF-Tert), (**C**) immortalized epithelial lung cells (BEAS-2B), and (**D**) breast adenocarcinoma cells (MDA-MB-231) using MTS reagent. Tested concentration range of EO was 125–1000 mg/L.

The cytotoxic activity of seven *S. officinalis* Eos was evaluated using MTS assay on four cell lines: normal dermal fibroblasts (HF77FA), immortalized human dermal fibroblasts (HDF-Tert), immortalized epithelial lung cells (BEAS-2B), and breast adenocarcinoma cells (MDA-MB-231). Results are presented in Figure 3. IC_50_ values for all four cell lines are presented in Table 3.

Statistical analysis revealed that the HDF-Tert cell line was most resistant towards *S. officinalis* EO’s (Tukey’s multiple comparisons test, *p* < 0.0001) followed by HF77FA and MDA-MB-231 cell lines with statistical difference between them (Tukey’s multiple comparisons test, *p* > 0.05). BEAS-2B cells were the least resistant to treatment compared to other cell lines (Tukey’s multiple comparisons test, *p* < 0.0001). The US National Cancer Institute defines four levels of cytotoxic activity: (1) high cytotoxic activity (IC_50_ < 20 mg/L); (2) moderate cytotoxic activity (IC_50_ = 21–200 mg/L); (3) weak cytotoxic activity (IC_50_ = 201–500 mg/L); and (4) no cytotoxic activity (IC_50_ > 500 mg/L) [40]. According to the mentioned criteria, *S. officinalis* EO’s did not show cytotoxic effect on the normal dermal fibroblast cell line (HF77FA). The exception is the sample SO-LO, which caused weak cytotoxic activity. The tested oils had no cytotoxic effect on the immortalized human dermal fibroblast cell line (HDF-Tert). The exact IC_50_ value was not determined for the SO-LA sample because it was outside the concentration interval in which the cells were treated. All seven EO’s showed weak cytotoxic activity against the BEAS-2B cell line. Two samples, SO-SP and SO-LO, show weak cytotoxic activity on the breast adenocarcinoma cell line (MDA-MB-231), while the other samples were not cytotoxic.

The studied *S. officinalis* EO’s contain compounds that have been proven to reduce the viability of cancer cells. Recent research has revealed the anticarcinogenic potential of α-thujone that inhibits the proliferation of glioblastoma cells, stimulates the anticarcinogenic immune response and inhibits melanoma metastasis [41]. Research conducted with human fetal lung fibroblasts (MRC-5), colorectal carcinoma (HT-29 and HCT116) cell lines, showed that camphor and 1,8-cineole have a similar cytotoxic effect [42]. According to the literature, 1,8-cineole is a potential anticarcinogenic agent against various types of cancer. The mechanism of action of 1,8-cineole is based on the activation of apoptosis via the p53 protein signaling pathway, caspases cleavage, mitochondrial stress activation, MAPK, and AKT signaling pathway activation [43]. Viridiflorol has been shown to induce early apoptosis and thus cause reduced viability of the MCF-7 breast cancer cell line [44]. In our work, the cells were treated with EO’s, a mixture of chemical compounds with different structures. Therefore, the synergistic effect of the aforementioned compounds and other identified compounds should be considered.

### 2.4. Statistical Correlation of Cytotoxicity and Essential Oil Chemical Composition

Statistical analysis was used to correlate the IC_50_ values of EO’s with chemical constituents, the results of the analysis are presented in Table 4. By calculating Pearson’s correlation coefficients, we have extrapolated which compounds or groups of compounds had higher or lower effect on the EO’s cytotoxicity for each cell line.

Statistical analysis showed a negative correlation between diterpene content and cell viability in the tests performed with HF77FA and BEAS-2B cell lines. According to a statistical test, EOs with more diterpenes were more cytotoxic. Verification of the truth of statistics can be done based on experimental observations. The highest amount of diterpenes was detected in SO-LO (3.8%) and SO-MA (2.6%) samples. Both samples were the most cytotoxic in the test performed with the BEAS-2B line, while the SO-LO sample showed the highest cytotoxic activity in the test with the HF77FA line. The increased cytotoxic activity can probably be attributed to the action of manool since it is the only diterpene compound detected in the analyzed sage EO’s.

The absence of more potent cytotoxic activity for sample SO-MA, which compared to other samples (SO-SP, SO-LA, SO-HV, SO-SV, SO-KO) contains a higher proportion of manool, can probably be attributed to the influence of camphor. A significant and positive correlation was observed between the amount of camphor and cytotoxic activity. The sample SO-KO (33.3%), which contains the highest amount of camphor, was the least cytotoxic to the HF77FA line and had one of the milder effects on the other cell lines as well. An agreement between experiment and statistics were observed for manool and camphor.

We have applied multiple classification strategies to represent the chemical composition of the obtained oils. With traditional classification according to the number of isoprene units and functional groups, we included unconventional classifications such as the presence of oxygen atom(s), carbon chain structure, and unsaturated bonds. This was done to improve the statistical analysis when comparing the chemical composition with the cytotoxic activity of the EOs.

According to the number of oxygen atoms in the molecule, compounds are classified into three groups: (1) compounds that do not contain an oxygen atom; (2) compounds containing one oxygen atom; and (3) compounds containing two oxygen atoms. The most represented number of compounds in all samples contain one oxygen atom in the molecule (71.6–84.8%). Statistical analysis revealed that compounds with one oxygen atom significantly increase the cytotoxicity of EOs. In contrast, compounds without oxygen atoms reduced EOs cytotoxicity in the HDF-Tert cell line. Ketone compounds (47.2–63.9%) are the most represented oxygenated compounds. No significant correlation was observed between the amount of ketone compounds and cytotoxic activity in tests performed in all cell lines, which could be explained by the presence of camphor in this subcategory (alongside thujones) for which we showed a correlation with lower cytotoxicity.

In the test performed with the HDF-Tert line, a high and positive correlation coefficient was observed for monoterpene hydrocarbons. It can be assumed that a higher amount of monoterpene hydrocarbons decreases the cytotoxic activity of the tested sage EOs. The highest amount of monoterpene hydrocarbons was detected in the SO-KO (16.8%) sample, which was the least cytotoxic EO for this cell line. The SO-SP, which was the second most cytotoxic EO, contained minor monoterpene hydrocarbons (9.2%). Another high and positive correlation coefficient was observed for alkene compounds. Again, the highest amount of alkene compounds was observed in the SO-KO sample (21.4%), while a minuscule amount was found in SO-SP (10.3%). The most interesting correlation was observed in the presence of oxygen atoms in the chemical compounds and IC_50_ value–solid, positive correlation for non-oxygenated compounds, and solid and negative correlation between mono-oxygenated compounds. Again, camphor had a solid positive correlation meaning it reduced the cytotoxicity of EO. For HDF/Tert cell line camphene, limonene and borneol also correlated with lower cytotoxicity. The sample SO-LA was not included in the statistical analysis as exact IC_50_ value could not be obtained.

In the test with the MDA-MB-231 line, a significant and negative correlation was observed between the amount of compounds built from multiple structural rings (n > 2) and cytotoxic activity. According to the statistical test, a higher amount of structurally more complex compounds increases the cytotoxic activity of sage EO’s. In EO’s samples SO-SP (9.3%) and SO-LO (9.2%), the highest amount of compounds with more than two structural rings was detected. The two aforementioned samples were the most cytotoxic to the MDA-MB-231 line, which is experimental evidence for a significant and negative correlation. The increased cytotoxic activity is probably caused by the tricyclic compound viridiflorol, for which a significant and negative correlation was observed.

In the test conducted with BEAS-2B and MDA-MB-231 cell lines, a significant and positive correlation was observed between the content of *α*-terpinolene and cytotoxic activity. According to statistics, sage oils containing a higher amount of *α*-terpinolene should be less cytotoxic on the two mentioned cell lines. Experimentally, samples containing α-terpinolene, SO-LA (0.4%) and SO-SV (0.3%) were the least cytotoxic on both cell lines.

Camphene [45] induced apoptosis by the intrinsic pathway in melanoma cells, while limonene [46] uses similar way of action, apoptosis, to induce cytotoxicity on human bladder cancer cell (T24 cell line). Camphor [47] is well-investigated on its anticancer activity tested on several human cancer cell lines, and borneol [48] together with α-terpinolene [49], viridiflorol [44] and manool [50] have some promising anticancer activity. As we could see, all of the individual compounds that were significantly correlated with increased or decreased cytotoxicity in our study were previously tested as pure compounds by other authors and proved to have promising anticancer activity. This could indicate that more potent cytotoxic activity could be expected from all tested *S. officinalis* EOs. The cytotoxic activity of EOs could result from synergistic or antagonistic reactions of chemical components present in the EOs. Further studies should bring more insight into the pharmacological interaction of the mixtures of chemical components and cells, both healthy and cancers.

## 3. Materials and Methods

### 3.1. Plant Material Origin

Wild grown plants of *Salvia officinalis* L. were collected in natural habitat according to legal and botanical regulations regarding wild plants harvesting. The authentication of plant material was carried out by means of macroscopic traits. Aerial parts of sage were collected in August and September 2018 in coastal area of Dalmatia region in Croatia. Voucher specimens (2018_SOfficinalis_SO) of plant materials used for this study have been deposited, with the date and location of collection, in the herbarium at the Department of Biochemistry, Faculty of Chemistry and Technology, Split, Croatia. The plant name was checked with http://www.theplantlist.org (accessed on 13 January 2023). The list of location coordinates is shown in Table 5. The plant material was dried for 15 days at room temperature and in the dark. Dried plant material was cut into smaller pieces and used for distillation.

### 3.2. Scanning Electron Microscopy

For observations of the epidermis surface, small pieces of *S. officinalis* leaf and stem were fixed in standard solution of 25mM phosphate buffer (pH = 7) with 3% glutaraldehyde (Sigma Aldrich, Munich, Germany), and 0.01% Tween 20. The samples were kept overnight in the same fixative solution without Tween 20. Another fixative used with the same procedure was FAA fixative (50% ethanol, 35% distilled water, 10% formaldehyde, and 5% glacial acetic acid). After overnight fixation at 4 °C samples were washed in phosphate buffer three times at 10-min intervals. The fixed plant material was dehydrated in graded ethanol series (30%, 50%, 70%, and 95%) and immersed in absolute ethanol (Sigma Aldrich, Munich, Germany). Ethanol was evaporated and fixed samples were further dried using critical-point dried in liquid CO_2_ using a K 850 Critical Point Dryer and sputter-coated with gold (10 mm thickness) using Q1 50R ES (Quorum, Lewes, UK). The observations were carried out using a FE–SEM Tescan Mira III (Tescan, Brno, Czech Republic) at an accelerating voltage of 4 kV [51,52].

### 3.3. Essential Oils Extraction, Gas Chromatography-Mass Spectrometry (GC-MS) Analyses

The essential oils were extracted from air-dried aerial parts of *Salvia officinalis* L. by performing hydrodistillation in Clevenger apparatus for 3 h and stored in a sealed vial, under 4 °C until use. The chemical composition of isolated EOs was determined using an instrumental technique combining a gas chromatograph (model 7890A, Agilent Technologies, Santa Clara, CA, USA) and a selective mass detector (model 5975C, Agilent Technologies). The temperature of the injector was 250 °C. The separation of the compounds was performed in a non-polar, capillary HP-5MS column in which the stationary phase consists of 5% phenyl-methylpolysiloxane (30 m × 0.25 mm, layer thickness 0.25 µm, Agilent Technologies). The carrier gas was helium applied at a flow rate of 1 mL/min. The oven temperature was programmed as follows: isothermal for 3 min at 70 °C, then increased to 200 °C at a rate of 3 °C/min and held isothermal for 20 min at 200 °C. The mass detector conditions were set: inlet temperature was 280 °C, ionization energy 70 eV, and scanning was performed in the mass range *m/z* 30–300.

The identification of individual chemical compounds was carried out based on retention indices (KI) determined in relation to the retention time of *n*-alkanes (C_9_–C_25_) and by comparing experimental mass spectra with spectra from the commercial Wiley 9 database (Wiley, New York, NY, USA) with an internal database created during previous analyses, and literature retention indices using NIST 2002 (National Institute of Standards and Technology, Gaithersburg, MD, USA) and NIST 14 mass spectra library, (D-Gaithersburg) Mass Spectral Libraries [53].

### 3.4. Cytotoxicity Assay

The cytotoxicity of EO was determined by performing MTS assay (CellTiter 96 Aqueous One Solution Cell Proliferation Assay; Promega, Madison, WI, USA) in normal dermal fibroblasts (HF77FA, Axol, UK), immortalized human dermal fibroblasts (HDF-Tert), immortalized lung epithelial cells (BEAS-2B) and breast adenocarcinoma cells (MDA-MB-231) obtained from Mediterranean Institute for Life Sciences (MedILS, Split, Croatia). Cells were grown in Dulbecco’s Modified Eagle Medium (DMEM, D5796, Sigma Aldrich, Munich, Germany), supplemented with 10% fetal bovine serum (FBS, 10500064, Thermo Fisher, Waltham, MA, USA) and 1% Antibiotic-Antimycotic (15240062, Thermo Fisher). For growing MDA-MB-231, 1% sodium pyruvate (NPY-B, Capricorn Scientific, Ebsdorfergrund, Germany) was added to the medium. The cells were grown in a humidified incubator at 37 °C and 5% CO_2_. For stock solutions preparation, EOs were dissolved in dimethyl sulfoxide (DMSO). When preparing working solutions, stock solutions were dissolved in the cell culture growth medium in a range of 125–1000 mg/L (DMSO < 1%). The treatment with EOs was performed 24h after seeding cells on 96-well plates. The cells were incubated for 48 h. Afterward, the old medium was replaced with the new one containing MTS reagent. After 4 h of incubation, the absorbance of samples (in biological and technical triplicates) was measured at a wavelength of 492 nm using a plate reader (EnSight Multimode Plate Reader, PerkinElmer, Waltham, MA, USA).

### 3.5. Statistical Analysis

Statistical analysis was performed using the GraphPad Prism software (version 9). Normalized cell viability curves were fitted using [Inhibitor] vs. normalized response—Variable slope in-built equation provided in the software. Calculated IC50 values are expressed as mean ± standard deviation. Data distribution was determined by performing Shapiro-Wilk normality test. Data were normally distributed (*p* > 0.05). Comparisons between EOs were performed using ANOVA test and analyzed post hoc with Tukey’s multiple comparisons tests. By calculating Pearson’s correlation coefficients *r*, the linear relationship between GC/MS results describing the chemical composition of sage EOs samples and IC_50_ values evaluating their cytotoxic activity was tested (Table 4). To interpret correlation results *r* values were used. Values 0 > *r* > −1 show a negative correlation meaning a higher amount of the constituent in the oil composition lower the IC_50_ value—constituent contributes to higher EO toxicity. Values 0 < *r* < 1 show a positive correlation meaning a higher amount of the constituent in the oil composition higher the IC_50_ value—constituent contributes to lower EO toxicity. The higher the *r* value deviates from 0 the stronger the correlation. Only results with *p* < 0.05 were deemed significant.

## 4. Conclusions

In this study, we investigated the stem and leaf tissue structures of *S. officinalis* using SEM, following two protocols that preserve the natural structure of the tissue surface. We also analyzed the chemical composition of seven *S. officinalis* EOs using GC/MS and assessed cell viability using MTS assay at various doses of EOs on healthy and cancerous cells.

The chemical analysis of seven *S. officinalis* EOs showed that they contain varying percentages of common chemical constituents: *α*- and *β*-thujone, camphor, and 1,8-cineole. The biological effects of sage EOs on cell viability were tested on different healthy and cancerous cells. The results showed that skin and immortalized cells were more resistant to sage EO, while the BEAS-2B cell line was the least resistant compared to the other cell lines. Using one cancerous and three healthy cell lines, we investigated the cytotoxic effect of our EOs to get results that simulate physiological conditions. Our results showed that two fibroblast and cancer-derived cell lines were less susceptible to *S. officinalis* EO than non-cancer cell lines derived from lung tissue. Healthy cell lines were more resistant to cytotoxic agents than cancer cell lines.

The statistical analysis and correlation of the chemical composition of EO and the tested cell viability have shown that the different cytotoxic activities of *S. officinalis* EOs are related to the differences in chemical composition.

To the best of our knowledge, this is the first study on the cytotoxic activity of *S. officinalis* EOs on BEAS-2B, HF77FA, and HDF-Tert cell lines. The data presented on the chemical composition of the EOs and the cytotoxic effect on 4 types of human cells support the pharmacotherapeutic potential that this plant is known to possess. High IC_50_ values of EOs tested on healthy human dermal fibroblasts give more arguments to the traditional use of S. officinalis EOs for skin treatments and cosmetic purposes. Further investigation of biological activities of mixtures of isolated chemical constituents and/or combinations of chemical isolated constituents, present in *S. officinalis* essential oils, could provide a more in depth understanding of the pharmacological properties of this plant.

## Figures and Tables

**Table 1:** Chemical composition of *S. officinalis* essential oils. The amount of compounds is expressed in %.

Compounds	KI	SO-SP	SO-LA	SO-HV	SO-SV	SO-MA	SO-LO	SO-KO	ID
*α*-Pinene	942	1.5	2.1	3.1	1.4	2.8	3.5	3.3	KI, MS
Camphene	959	3.8	2.9	2.1	3.7	5.0	2.5	7.4	KI, MS
*β*-Pinene	985	1.0	1.3	0.4	0.8	0.9	1.1	1.8	KI, MS
*β*-Myrcene	994	0.5	0.6	0.7	0.8	0.8	0.7	0.7	KI, MS
*p*-Cymene	1031	0.8	0.8	1.3	0.9	1.0	1.0	0.6	KI, MS
Limonene	1035	1.6	1.4	1.8	2.3	2.3	1.8	3.0	KI, MS
*γ*-Terpinene	1065	0.0	0.3	0.2	0.2	0.0	0.3	0.0	KI, MS
*α*-Terpinolene	1092	0.0	0.4	0.0	0.3	0.0	0.0	0.0	KI, MS
1,8-Cineole	1039	8.1	7.2	4.2	9.9	8.6	9.8	9.8	KI, MS
Linalool	1103	0.0	0.0	0.4	0.4	0.4	0.3	0.6	KI, MS
*α*-Thujone (cis)	1111	26.5	32.5	42.6	25.3	27.3	33.1	12.6	KI, MS
*β*-Thujone (trans)	1112	13.8	9.5	7.4	12.2	6.7	3.7	1.3	KI, MS
Camphor	1150	23.6	20.5	12.4	22.1	18.2	13.3	33.3	KI, MS
Borneol	1172	3.5	3.2	2.6	4.0	3.3	3.8	5.7	KI, MS
Terpinen-4-ol	1182	0.0	0.8	0.8	0.6	0.5	0.7	0.6	KI, MS
*p*-Cymen-8-ol	1191	0.0	0.1	0.9	0.0	0.0	0.2	0.0	KI, MS
*α*-Terpineol	1195	0.0	0.2	0.2	0.0	0.0	0.2	0.0	KI, MS
Bornyl acetate	1288	3.3	0.2	1.0	2.3	1.4	1.9	3.3	KI, MS
Thymol	1302	0.0	0.2	0.0	0.0	0.0	0.0	0.0	KI, MS
Carvacrol	1312	0.0	0.0	0.2	0.0	0.0	0.2	0.0	KI, MS
*α*-Humulene	1456	1.8	2.0	2.3	2.8	2.2	3.0	3.6	KI, MS
Viridiflorol	1593	9.3	6.4	8.2	5.3	5.2	8.5	7.7	KI, MS
*trans-β*-Caryophyllene	1422	0.0	0.7	0.1	0.3	0.0	1.1	0.7	KI, MS
Caryophyllene oxide	1584	0.0	0.7	0.1	0.0	0.0	0.7	0.0	KI, MS
Manool	2052	0.0	0.0	0.0	1.2	2.6	3.8	0.0	KI, MS
TOTAL		99.1	94.0	93.0	96.8	89.2	95.2	96.0	
Yield		0.56	0.91	1.58	2.14	1.58	0.65	1.93	

KI = Kovats retention index determined on a HP-5MS column using the homologous series of *n*-alkanes (C_9_–C_25_); MS = mass spectra; SO—*Salvia officinalis* essential oil; locations (SP, LA, HV, SV, MA, LO, KO).

**Table 2:** Classification of identified compounds according to different groups. The amount of compounds for each group in EO’s is expressed in %.

**Terpene Compounds**	**SO-SP**	**SO-LA**	**SO-HV**	**SO-SV**	**SO-MA**	**SO-LO**	**SO-KO**
Monoterpene hydrocarbons	9.2	9.8	9.6	10.4	12.8	10.9	16.8
Derivatives of monoterpenes	78.8	74.4	72.7	76.8	66.4	67.2	67.2
Sesquiterpenes	11.1	9.8	10.7	8.4	7.4	13.3	12.0
Diterpene	0.0	0.0	0.0	1.2	2.6	3.8	0.0
**Functional Groups**	**SO-SP**	**SO-LA**	**SO-HV**	**SO-SV**	**SO-MA**	**SO-LO**	**SO-KO**
Alkenes	10.2	11.7	10.7	12.6	14.0	14.0	20.5
Alcohols	12.8	10.6	12.2	11.5	12.0	17.3	14.6
Ketones	63.9	62.5	62.4	59.6	52.2	50.1	47.2
Aromatic	0.8	1.1	2.4	0.9	1.0	1.4	0.6
Others	11.4	8.1	5.3	12.2	10.0	12.4	13.1
**Structure**	**SO-SP**	**SO-LA**	**SO-HV**	**SO-SV**	**SO-MA**	**SO-LO**	**SO-KO**
Acylic (n = 0)	0.5	0.6	1.1	1.2	1.2	1.0	1.3
Cyclic (n = 1)	4.2	6.2	7.7	7.1	6.0	7.4	7.8
Cyclic (n = 2)	85.1	80.1	75.9	83.2	76.8	77.6	79.2
Cyclic (n > 2)	9.3	7.1	8.3	5.3	5.2	9.2	7.7
**Number of Oxygen Atoms**	**SO-SP**	**SO-LA**	**SO-HV**	**SO-SV**	**SO-MA**	**SO-LO**	**SO-KO**
n = 0	11.0	12.5	12.0	13.5	15.0	15.0	21.1
n = 1	84.8	81.3	80.0	81.0	72.8	78.3	71.6
n = 2	3.3	0.2	1.0	2.3	1.4	1.9	3.3
**Number of Unsaturated Bonds in Molecules**	**SO-SP**	**SO-LA**	**SO-HV**	**SO-SV**	**SO-MA**	**SO-LO**	**SO-KO**
n = 0	20.9	16.8	15.0	19.2	17.1	22.1	23.2
n = 1	73.5	70.7	70.1	68.4	62.8	60.7	63.6
n = 2	1.6	2.8	2.5	4.7	5.3	7.3	4.3
n = 3	2.3	2.6	3.0	3.6	3.0	3.7	4.3
Ar *	0.8	1.1	2.4	0.9	1.0	1.4	0.6

* Ar = compounds with aromatic ring.

**Table 3:** Estimated IC_50_ = mean ± standard deviation (mg/L) values after treatment cells with *S. officinalis* essential oils.

Samples	HF77FA	HDF-Tert	BEAS-2B	MDA-MB-231
SO-SP	511.00 ± 34.77	819.00 ± 1.73	279.00 ± 80.61	423.00 ± 30.81
SO-LA	732.00 ± 31.32	>1000	408.00 ± 6.24	618.67 ± 136.14
SO-HV	585.33 ± 49.14	853.00 ± 5.20	347.67 ± 33.01	514.33 ± 94.84
SO-SV	669.00 ± 12.12	828.50 ± 122.33	363.67 ± 1.15	778.00 ± 67.51
SO-MA	539.00 ± 46.51	839.33 ± 15.50	218.00 ± 14.11	535.00 ± 122.14
SO-LO	419.00 ± 25.51	817.33 ± 7.02	229.00 ± 33.78	446.67 ± 67.28
SO-KO	784.00 ± 7.00	954.50 ± 57.28	350.00 ± 25.36	618.00 ± 113.50

**Table 4:** The results of statistical correlation analysis for cytotoxic activity and chemical composition of *S. officinalis* EO’s. Only statistically significant results (*p* < 0.05) were presented in the table. *r*—Pearson’s correlation coefficients; *p*—calculated confidence level; ns—not statistically significant.

**Terpene Compounds vs. IC_50_**	**HF77FA**	**HDF-Tert**	**BEAS-2B**	**MDA-MB-213**
Monoterpene hydrocarbons	ns	*r* = 0.8789 *p* = 0.0106	ns	ns
Derivatives of monoterpenes	ns	ns	ns	ns
Sesquiterpenes	ns	ns	ns	ns
Diterpene	*r* = −0.6704 *p* = 0.0497	ns	*r* = −0.7589 *p* = 0.0239	ns
Unidentified	ns	ns	ns	ns
**Functional Groups vs. IC_50_**	**HF77FA**	**HDF-Tert**	**BEAS-2B**	**MDA-MB-213**
Alkenes	ns	*r* = 0.8327 *p* = 0.0198	ns	ns
Alcohols	ns	ns	ns	ns
Ketones	ns	ns	ns	ns
Aromatic	ns	ns	ns	ns
Other compounds	ns	ns	ns	ns
**Structure vs. IC_50_**	**HF77FA**	**HDF-Tert**	**BEAS-2B**	**MDA-MB-213**
Acylic (n = 0)	ns	ns	ns	ns
Cyclic (n = 1)	ns	ns	ns	ns
Cyclic (n = 2)	ns	ns	ns	ns
Cyclic (n > 2)	ns	ns	ns	*r* = −0.7736 *p* = 0.0206
**Number of Oxygen Atoms vs. IC_50_**	**HF77FA**	**HDF-Tert**	**BEAS-2B**	**MDA-MB-213**
n = 0	ns	*r* = 0.8276 *p* = 0.0210	ns	ns
n = 1	ns	*r* = −0.8485 *p* = 0.0163	ns	ns
n = 2	ns	ns	ns	ns
**Number of Unsaturated Bonds vs. IC_50_**	**HF77FA**	**HDF-Tert**	**BEAS-2B**	**MDA-MB-213**
n = 0	ns	ns	ns	ns
n = 1	ns	ns	ns	ns
n = 2	ns	ns	ns	ns
n = 3	ns	ns	ns	ns
Ar	ns	ns	ns	ns
**Individual Compounds vs. IC_50_**	**HF77FA**	**HDF-Tert**	**BEAS-2B**	**MDA-MB-213**
Camphene	ns	*r* = 0.7848 *p* = 0.0322	ns	ns
Limonene	ns	*r* = 0.8069 *p* = 0.0262	ns	ns
Camphor	*r* = 0.7044 *p* = 0.0386	*r* = 0.7435 *p* = 0.0451	ns	ns
Borneol	ns	*r* = 0.7823 *p* = 0.0330	ns	ns
trans-β-Caryophyllene	ns	ns	ns	ns
*Caryophyllene oxide*	ns	ns	ns	ns
α-Terpinolene	Ns	ns	*r* = 0.7028 *p* = 0.0391	*r* = 0.6844 *p* = 0.0449
Viridiflorol	ns	ns	ns	*r* = −0.7764 *p* = 0.0200
Manool	ns	ns	*r* = −0.7706 *p* = 0.0213	ns

**Table 5:** The locations of *S. officinalis* plants sampling.

Name of Location	Latitude	Longitude	Approximate Elevation
Sipan (SO-SP)	42°43′25.26″ N	17°52′34.21″ E	57 m
Lastovo (SO-LA)	42°45′4.93″ N	16°52′36.64″ E	102 m
Hvar (SO-HV)	43°10′23.18″ N	16°26′18.28″ E	37 m
Seget Vranjica (SO-SV)	43°30′50.37″ N	16°11′13.29″ E	19 m
Marina (SO-MA)	43°30′37.31″ N	16°7′35.36″ E	8 m
Lozovac (SO-LO)	43°48′1.90″ N	15°57′36.18″ E	183 m
Kornati (SO-KO)	43°49′46.18″ N	15°16′17.12″ E	16 m

## Data Availability

Not applicable.
